# Synthesis and Anticancer Activity of *N*-Aryl-5-substituted-1,3,4-oxadiazol-2-amine Analogues

**DOI:** 10.1155/2014/814984

**Published:** 2014-05-26

**Authors:** Mohamed Jawed Ahsan, Jyotika Sharma, Monika Singh, Surender Singh Jadav, Sabina Yasmin

**Affiliations:** ^1^Department of Pharmaceutical Chemistry, Maharishi Arvind College of Pharmacy, Jaipur, Rajasthan 302 023, India; ^2^Department of Pharmaceutical Chemistry, Birla Institute of Technology, Mesra, Ranchi, Jharkhand 835 215, India

## Abstract

In continuance of our search for anticancer agents, we report herein the synthesis and anticancer activity of some novel oxadiazole analogues. The compounds were screened for anticancer activity as per National Cancer Institute (NCI US) protocol on leukemia, melanoma, lung, colon, CNS, ovarian, renal, prostate, and breast cancers cell lines. *N*-(2,4-Dimethylphenyl)-5-(4-methoxyphenyl)-1,3,4-oxadiazol-2-amine (**4s**) showed maximum activity with mean growth percent (GP) of 62.61 and was found to be the most sensitive on MDA-MB-435 (melanoma), K-562 (leukemia), T-47D (breast cancer), and HCT-15 (colon cancer) cell lines with GP of 15.43, 18.22, 34.27, and 39.77, respectively. Maximum GP was observed on MDA-MB-435 (melanoma) cell line (GP = 6.82) by compound *N*-(2,4-dimethylphenyl)-5-(4-hydroxyphenyl)-1,3,4-oxadiazol-2-amine (**4u**).

## 1. Introduction

An estimated 14.1 million cancer cases and 8.2 million deaths occurred globally in 2012, and the annual new cases will jump to 19.3 million by 2025. Cancer deaths were up to nearly 8 percent from 7.6 million in a previous survey in 2008. An urgent need in cancer control today is to develop effective and affordable approaches to the early detection, diagnosis, and treatment of cancer. Tobacco use is the greatest single avoidable risk factor for cancer mortality worldwide causing an estimated 22% of cancer deaths per annum. 22% of mouth and oropharynx cancers in men are attributable to alcohol. Almost 22% of cancer deaths in the developing world and 6% in industrialized countries are due to infectious agents and environmental pollution of air, water, and soil with carcinogenic chemicals accounts for 1–4% of all cancers. Residential exposure to radon gas from soil and building materials is estimated to cause 3–14% of all lung cancers, making it the second cause of lung cancer after tobacco smoke. Ultraviolet (UV) radiation, in particular solar radiation, is carcinogenic to humans, causing all major types of skin cancer, which includes basal cell carcinoma, squamous cell carcinoma, and melanoma. Breast cancer is the most frequently diagnosed cancer and the leading cause of cancer death among females, accounting for 23% of the total cancer cases and 14% of the cancer deaths [1–3]. The therapeutic approach of cancer includes chemotherapy, radiotherapy, surgery, immunotherapy, monoclonal antibody therapy, hormonal therapy, targeted therapy, and angiogenesis inhibition. The drugs used for the treatment of cancer are generally cytotoxic, and their use is often coupled with various adverse effects including bone marrow depression, alopecia, and drug induced cancer. Resistance, cytotoxicity, and genotoxicity of anticancer drugs are the reasons that warrant the search for newer anticancer agents, and researchers from various laboratories throughout the world are ardently engaged to find a more pleasant solution for the treatment of cancer.

The widespread use of 1,3,4-oxadiazoles as a scaffold in medicinal chemistry establishes this moiety as a member of the privileged structural class due to their remarkable biological and pharmacological properties, such as anticancer [4–7], antitubercular [8, 9], antibacterial [8], antifungal [10], anti-HIV [11], anti-inflammatory [12], and insecticidal [13] activities. Zibotentan, an endothelin receptor A (ET_A_) antagonist, is an anticancer agent which contains 1,3,4-oxadiazole ring [14]. Inspired by all these facts, we have designed based on the molecular properties prediction by Molinspiration and toxicity risk prediction by Osiris software and synthesized oxadiazole analogues for anticancer screening [15, 16]. The number of rotatable bonds (NROTB) and Lipinski's rule of five were also calculated [17]. The rule of five states that most molecules with good membrane permeability have log* P* (partition coefficient) ≤ 5, molecular weight (MW) ≤ 500, number of hydrogen bond acceptors ≤ 10, and number of hydrogen bond donors ≤ 5. This rule is widely used as a filter for drug-like properties. Furthermore, none of the compounds violated Lipinski's parameters, making them potentially promising agents. The pharmacokinetic parameters important for good oral bioavailability of* N*-aryl-5-substituted-1,3,4-oxadiazol-2-amine analogues (**4a**–**x**) are given in Table 1, and the toxicity risk prediction (mutagenic, irritant, and reproductive effect) calculated with Osiris is given in Table 2. The toxicity risk prediction showed that all these oxadiazoles are comparatively less toxic than the standard drug fluorouracil and methotrexate. Good intestinal absorption, reduced molecular flexibility (measured by the number of rotatable bonds), low polar surface area, and total hydrogen bond count (sum of donors and acceptors) are important predictors of good oral bioavailability [18, 19]. Membrane permeability and bioavailability are always associated with some basic molecular descriptors such as log* P* (partition coefficient), molecular weight (MW), or hydrogen bond acceptors and donors counts in a molecule. The number of rotatable bonds is important for conformational changes of molecules under study and ultimately for the binding with receptors or channels. It is revealed that, for passing oral bioavailability criteria, the number of rotatable bond should be ≤10 [18]. In the present studies the title compounds have log* P* value varied from 2.73 to 4.72 (<5), MW varied from 203 to 375 (<500), number of hydrogen bond acceptors varied from 4 to 6 (≤10), number of hydrogen bond donors varied from 1 to 2 (≤5), and number of rotatable bond varied from 3 to 5 (≤10).

## 2. Materials and Methods

### 2.1. Chemistry

All chemicals were procured from E Merck, CDH Drug laboratory, and SD Fine Chemicals. Melting points were determined by open tube capillary method and were uncorrected. Purity of the compounds was checked by elemental analysis, and the progress of reactions was monitored by TLC plates (silica gel G) using mobile phase, chloroform : methanol (9 : 1) and acetone : n-hexane (8 : 2), and the spots were identified by iodine vapours or UV light. IR spectra were recorded on a Shimadzu 8201 PC, FT-IR spectrometer (KBr pellets). NMR spectra were recorded on a Bruker AC 300 MHz spectrometer using TMS as internal standard in DMSO* d*_*6*_. Mass spectra were recorded on a Bruker Esquire LCMS using ESI, and elemental analyses were performed on Perkin-Elmer 2400 Elemental Analyzer.

### 2.2. General Method for the Synthesis of Substituted Phenyl Urea Analogues (**2a–d**)

Aromatic anilines (0.1 mol) were dissolved in 20 mL of glacial acetic acid and 10 mL of hot water, and sodium cyanate (6.5 g, 0.1 mol) in 80 mL of hot water was added with stirring. It was allowed to stand for 30 min, then cooled in ice bath, filtered with suction, dried, and recrystallized from boiling water to obtain substituted phenyl urea (**2a–d**) [20–22].

### 2.3. General Method for the Synthesis of Semicarbazide Analogues (**3a–d**)

Equimolar quantities (0.05 mol) of substituted phenyl urea (**2a–d**) and hydrazine hydrate (AR 99-100%) (2.5 mL, 0.05 mol) in ethanol were refluxed for 48 h with stirring. The two-thirds volume of alcohol was distilled by vacuum distillation and then poured into the crushed ice. The resultant precipitate was filtered, washed with water, and dried. The solid mass was recrystallized from 50 mL absolute ethanol to obtain semicarbazide analogues (**3a–d**) [20–22].

### 2.4. General Method for the Synthesis of 5-Substituted-N-aryl-1,3,4-oxadiazol-2-amine Analogues (**4a–x**)

Substituted phenyl semicarbazide (0.005 mol) (**3a–d**) and aromatic aldehydes (0.005 mol) were refluxed for 10–12 h using 20 mol% NaHSO_3_ and ethanol-water system (1 : 2, v/v) solvent [23]. After completion of reaction, the excess solvent was removed and the concentrate was poured into crushed ice filter, washed with water, dried, and recrystallized with absolute ethanol to obtain the final product (**4a–x**). The reaction was monitored throughout by thin layer chromatography (TLC) using chloroform: methanol (9 : 1) and acetone : n-hexane (8 : 2) as mobile phase.

#### 2.4.1. N-(4-Methylphenyl)-5-(4-methoxyphenyl)-1,3,4-oxadiazol-2-amine (**4a**)

Yield 72%, Mp. 178–180°C; IR: (KBr) cm^−1^: 3219 (NH), 1523 (C=N), 1173 (C–O–C). ^1^H NMR (300 MHz, DMSO-*d*_6_): *δ* 2.25 (3H, s, CH_3_), 3.79 (3H, s, OCH_3_), 6.95–6.98 (2H, d,* J* = 7.8 Hz, ArH), 7.07–7.09 (2H, d,* J* = 7.2 Hz, ArH), 7.50–7.52 (2H, d,* J* = 7.5 Hz, ArH), 7.73–7.76 (2H, d,* J* = 7.8 Hz, ArH), 8.67 (1H, s, NH); ^13^C NMR (75 Hz, DMSO-*d*_6_): *δ* 24.31, 55.92, 114.82, 116.21, 118.55, 128.12, 128.52, 129.92, 140.09, 152.12, 160.71, 164.55;* m/z* = 281 (M^+^), 282 (M+1)^+^. Cal/Ana: [C (68.22) 68.31 H (5.45) 5.37 N (14.82) 14.94].

#### 2.4.2. N-(4-Methylphenyl)-5-(4-chlorophenyl)-1,3,4-oxadiazol-2-amine (**4b**)

Yield 68%, Mp. 214–216°C; IR: (KBr) cm^−1^: 3191 (NH), 1531 (C=N), 1203 (C–O–C), 6.94 (C–Cl). ^1^H NMR (300 MHz, DMSO-*d*_6_): *δ* 2.03 (3H, s, CH_3_), 6.82–6.85 (2H, d,* J* = 8.4 Hz, ArH), 7.08–7.12 (2H, d,* J* = 8.1 Hz, ArH), 7.21–7.24 (2H, d,* J* = 8.1 Hz, ArH), 7.31–7.34 (2H, d,* J* = 8.4 Hz, ArH), 8.44 (1H, s, NH);* m/z* = 285 (M^+^), 287 (M+2)^+^. Cal/Ana: [C (63.01) 63.05 H (4.19) 4.23 N (14.73) 14.71].

#### 2.4.3. N-(4-Methylphenyl)-5-(4-hydroxyphenyl)-1,3,4-oxadiazol-2-amine (**4c**)

Yield 74%, Mp. 182–185°C; IR: (KBr) cm^−1^: 3402 (OH), 3199 (NH), 1511 (C=N), 1119 (C–O–C), 766 (C–Cl). ^1^H NMR (300 MHz, DMSO-*d*_6_): *δ* 2.24 (3H, s, CH_3_), 6.78–6.81 (2H, d,* J* = 6.3 Hz, ArH), 7.06–7.08 (2H, d,* J* = 5.4 Hz, ArH), 7.49–7.51 (2H, d,* J* = 6 Hz, ArH), 7.62–7.64 (2H, d,* J* = 6.3 Hz, ArH), 8.62 (1H, s, NH), 10.36 (1H, s, OH);* m/z* = 267 (M^+^). Cal/Ana: [C (67.31) 67.40 H (4.86) 4.90 N (15.76) 15.72].

#### 2.4.4. N-(4-Methylphenyl)-5-(3,4-dimethoxyphenyl)-1,3,4-oxadiazol-2-amine (**4d**)

Yield 70%, Mp. 176–178°C; IR: (KBr) cm^−1^: 3212 (NH), 1521 (C=N), 1119 (C–O–C). ^1^H NMR (300 MHz, DMSO-*d*_6_): *δ* 2.24 (3H, s, CH_3_), 3.79 (6H, s, OCH_3_), 6.77–6.80 (2H, d,* J* = 6.4 Hz, ArH), 7.06–7.08 (2H, d,* J* = 5.4 Hz, ArH), 7.53–7.55 (2H, d,* J* = 6.1 Hz, ArH), 7.61 (1H, s, ArH), 8.44 (1H, s, NH); ^13^C NMR (75 Hz, DMSO-*d*_6_): *δ* 24.32, 56.21, 112.31, 115.81, 116.23, 119.51, 120.82, 128.42, 129.99, 140.19, 142.11, 149.81, 150.31, 164.51;* m/z* = 311 (M^+^). Cal/Ana: [C (65.41) 65.58 H (5.46) 5.55 N (13.76) 13.50].

#### 2.4.5. N-(4-Methylphenyl)-5-(2-furyl)-1,3,4-oxadiazol-2-amine (**4e**)

Yield 66%, Mp. 182–184°C; IR: (KBr) cm^−1^: 3219 (NH), 1523 (C=N), 1109 (C–O–C). ^1^H NMR (300 MHz, DMSO-*d*_6_): *δ* 2.23 (3H, s, CH_3_), 6.86–6.89 (2H, d,* J* = 6.1 Hz, ArH), 6.96–6.99 (2H, d,* J* = 5.4 Hz, ArH), 7.36–7.41 (3H, m, ArH), 8.33 (1H, s, NH);* m/z* = 241 (M^+^). Cal/Ana: [C (64.66) 64.72 H (4.56) 4.60 N (17.52) 17.42].

#### 2.4.6. N-(4-Methylphenyl)-5-ethyl-1,3,4-oxadiazol-2-amine (**4f**)

Yield 70%, Mp. 210–212°C; IR: (KBr) cm^−1^: 3222 (NH), 1529 (C=N), 1116 (C–O–C). ^1^H NMR (300 MHz, DMSO-*d*_6_): *δ* 1.32–1.35 (3H, t,* J* = 6.4 Hz, CH_3_), 2.24 (3H, s, CH_3_), 2.62 (2H, m, CH_2_), 6.89–6.92 (2H, d,* J* = 6.1 Hz, ArH), 6.93–6.96 (2H, d,* J* = 6.4 Hz, ArH), 8.62 (1H, s, NH);* m/z* = 203 (M^+^). Cal/Ana: [C (64.94) 65.01 H (6.46) 6.45 N (20.76) 20.68].

#### 2.4.7. N-(4-Bromophenyl)-5-(4-methoxyphenyl)-1,3,4-oxadiazol-2-amine (**4g**)

Yield 81%, Mp. 198–200°C; IR: (KBr) cm^−1^: 3212 (NH), 1521 (C=N), 1119 (C–O–C), 635 (C–Br). ^1^H NMR (300 MHz, DMSO-*d*_6_): *δ* 3.79 (3H, s, OCH_3_), 6.77–6.80 (2H, d,* J* = 6.1 Hz, ArH), 6.96–6.98 (2H, d,* J* = 5.1 Hz, ArH), 7.41–7.43 (2H, d,* J* = 6 Hz, ArH), 7.52-7.53 (2H, d,* J* = 6.6 Hz, ArH), 8.43 (1H, s, NH);* m/z* = 345 (M^+^), 347 (M^+^+2). Cal/Ana: [C (52.11) 52.04 H (3.46) 3.49 N (12.18) 12.14].

#### 2.4.8. N-(4-Bromophenyl)-5-(4-chlorophenyl)-1,3,4-oxadiazol-2-amine (**4h**)

Yield 70%, Mp. 177-178°C; IR: (KBr) cm^−1^: 3211 (NH), 1519 (C=N), 1112 (C–O–C), 643 (C–Br), 787 (C-Cl).^ 1^H NMR (300 MHz, DMSO-*d*_6_): *δ* 6.71–6.73 (2H, d,* J* = 6.2 Hz, ArH), 6.92–6.94 (2H, d,* J* = 6.0 Hz, ArH), 7.12–7.14 (2H, d,* J* = 6 Hz, ArH), 7.32–7.34 (2H, d,* J* = 6.1 Hz, ArH), 8.53 (1H, s, NH);* m/z* = 348 (M^+^), 350 (M^+^+2), 352 (M^+^+4). Cal/Ana: [C (47.91) 47.96 H (2.56) 2.59 N (11.96) 11.99].

#### 2.4.9. N-(4-Bromophenyl)-5-(4-hydroxyphenyl)-1,3,4-oxadiazol-2-amine (**4i**)

Yield 67%, Mp. 190–192°C; IR: (KBr) cm^−1^: 3402 (OH), 3192 (NH), 1525 (C=N), 1119 (C–O–C), 634 (C–Br). ^1^H NMR (300 MHz, DMSO-*d*_6_): *δ* 6.73–6.75 (2H, d,* J* = 6.1 Hz, ArH), 6.95–6.97 (2H, d,* J* = 5.1 Hz, ArH), 7.39–7.41 (2H, d,* J* = 6 Hz, ArH), 7.47–7.50 (2H, d,* J* = 6.6 Hz, ArH), 8.44 (1H, s, NH), 10.42 (1H, s, OH);* m/z* = 331 (M^+^), 333 (M^+^+2). Cal/Ana: [C (65.41) 65.58 H (5.46) 5.55 N (13.76) 13.50].

#### 2.4.10. N-(4-Bromophenyl)-5-(3,4-dimethoxyphenyl)-1,3,4-oxadiazol-2-amine (**4j**)

Yield 75%, Mp. 186–188°C; IR: (KBr) cm^−1^: 3218 (NH), 1513 (C=N), 1112 (C–O–C), 6.37 (C–Br). ^1^H NMR (300 MHz, DMSO-*d*_6_): *δ* 3.79 (6H, s, OCH_3_), 6.79–6.81 (2H, d,* J* = 6.2 Hz, ArH), 6.92–6.95 (2H, d,* J* = 6.1 Hz, ArH), 7.44–7.47 (2H, d,* J* = 6 Hz, ArH), 7.51 (1H, s, ArH), 8.42 (1H, s, NH); ^13^C NMR (75 Hz, DMSO-*d*_6_): *δ* 56.12, 112.33, 113.11, 115.83, 118.51, 119.12, 120.82, 132.53, 142.11, 149.08, 149.51, 162.02, 164.59;* m/z* = 375 (M^+^), 377 (M^+^+2). Cal/Ana: [C (51.11) 51.08 H (3.72) 3.75 N (11.16) 11.17].

#### 2.4.11. N-(4-Bromophenyl)-5-(2-furyl)-1,3,4-oxadiazol-2-amine (**4k**)

Yield 78%, Mp. 138–140°C; IR: (KBr) cm^−1^: 3212 (NH), 1501 (C=N), 1121 (C–O–C), 634 (C–Br). ^1^H NMR (300 MHz, DMSO-*d*_6_): *δ* 6.80–6.82 (2H, d,* J* = 6.2 Hz, ArH), 6.92–6.94 (2H, d,* J* = 5.1 Hz, ArH), 7.41–7.44 (3H, m, ArH), 8.14 (1H, s, NH);* m/z* = 305 (M^+^), 307 (M^+^+2). Cal/Ana: [C (47.01) 47.08 H (2.66) 2.63 N (13.76) 13.73].

#### 2.4.12. N-(4-Bromophenyl)-5-ethyl-1,3,4-oxadiazol-2-amine (**4l**)

Yield 70%, Mp. 178–180°C; IR: (KBr) cm^−1^: 3210 (NH), 1524 (C=N), 1116 (C–O–C), 634 (C–Br). ^1^H NMR (300 MHz, DMSO-*d*_6_): *δ* 1.34 (3H, s, CH_3_), 2.59 (2H, m, CH_2_), 7.33–7.35 (2H, d,* J* = 6.3 Hz, ArH), 7.56–7.58 (2H, d,* J* = 6.3 Hz, ArH), 8.66 (1H, s, NH);* m/z* = 267 (M^+^), 269 (M^+^+2). Cal/Ana: [C (44.79) 44.80 H (3.73) 3.76 N (15.65) 15.67].

#### 2.4.13. N-(4-Chlorophenyl)-5-(4-methoxyphenyl)-1,3,4-oxadiazol-2-amine (**4m**)

Yield 82%, Mp. 188–190°C; IR: (KBr) cm^−1^: 3211 (NH), 1523 (C=N), 1129 (C–O–C), 695 (C–Cl). ^1^H NMR (300 MHz, DMSO-*d*_6_): *δ* 3.83 (3H, s, OCH_3_), 6.96–6.98 (2H, d,* J* = 7.5 Hz, ArH), 7.31–7.33 (2H, d,* J* = 7.5 Hz, ArH), 7.68–7.71 (2H, d,* J* = 7.5 Hz, ArH), 7.78–7.81 (2H, d,* J* = 7.8 Hz, ArH), 8.61 (1H, s, NH);* m/z* = 301 (M^+^), 303 (M^+^+2). Cal/Ana: [C (59.66) 59.71 H (3.97) 4.01 N (13.98) 13.93].

#### 2.4.14. N-(4-Chlorophenyl)-5-(4-fluorophenyl)-1,3,4-oxadiazol-2-amine (**4n**)

Yield 71%, Mp. 185–187°C; IR: (KBr) cm^−1^: 3214 (NH), 1521 (C=N), 1115 (C–O–C), 787 (C–F), 694 (C–Cl).^ 1^H NMR (300 MHz, DMSO-*d*_6_): *δ* 7.21–7.24 (2H, d,* J* = 8.7 Hz, ArH), 7.31–7.34 (2H, d,* J* = 8.7 Hz, ArH), 7.67–7.70 (2H, d,* J* = 8.7 Hz, ArH), 7.89–7.92 (2H, d,* J* = 5.7 Hz, ArH), 8.65 (1H, s, NH);* m/z* = 289 (M^+^), 291 (M^+^+2). Cal/Ana: [C (58.08) 58.04 H (3.11) 3.13 N (14.46) 14.51].

#### 2.4.15. N-(4-Chlorophenyl)-5-(4-chlorophenyl)-1,3,4-oxadiazol-2-amine (**4o**)

Yield 72%, Mp. 162–164°C; IR: (KBr) cm^−1^: 3212 (NH), 1511 (C=N), 1102 (C–O–C), 699 (C–Cl).^ 1^H NMR (300 MHz, DMSO-*d*_6_): *δ* 7.27–7.29 (2H, d,* J* = 6.1 Hz, ArH), 7.39–7.41 (2H, d,* J* = 6.0 Hz, ArH), 7.62–7.64 (2H, d,* J* = 6.1 Hz, ArH), 7.81–7.83 (2H, d,* J* = 6.2 Hz, ArH), 8.05 (1H, s, NH);* m/z* = 305 (M^+^), 307 (M^+^+2). Cal/Ana: [C (54.85) 54.92 H (2.91) 2.96 N (13.86) 13.73].

#### 2.4.16. N-(4-Chlorophenyl)-5-(4-hydroxyphenyl)-1,3,4-oxadiazol-2-amine (**4p**)

Yield 69%, Mp. 140–142°C; IR: (KBr) cm^−1^: 3402 (OH), 3192 (NH), 1521 (C=N), 1118 (C–O–C), 697 (C–Cl). ^1^H NMR (300 MHz, DMSO-*d*_6_): *δ* 7.23–7.25 (2H, d,* J* = 6.1 Hz, ArH), 7.32–7.34 (2H, d,* J* = 6.2 Hz, ArH), 7.68–7.70 (2H, d,* J* = 6.1 Hz, ArH), 7.83–7.85 (2H, d,* J* = 5.7 Hz, ArH), 7.95 (1H, s, NH), 10.41 (1H, s, OH);* m/z* = 287 (M^+^), 289 (M^+^+2). Cal/Ana: [C (58.41) 58.45 H (3.46) 3.50 N (14.66) 14.61].

#### 2.4.17. N-(4-Chlorophenyl)-5-(3,4-dimethoxyphenyl)-1,3,4-oxadiazol-2-amine (**4q**)

Yield 76%, Mp. 170–172°C; IR: (KBr) cm^−1^: 3218 (NH), 1518 (C=N), 1116 (C–O–C), 699 (C–Cl). ^1^H NMR (300 MHz, DMSO-*d*_6_): *δ* 3.78 (6H, s, OCH_3_), *δ* 7.29–7.31 (2H, d,* J* = 6.1 Hz, ArH), 7.41–7.43 (2H, d,* J* = 6.1 Hz, ArH), 7.69–7.73 (2H, d,* J* = 7.2 Hz, ArH), 7.95 (1H, s, ArH), 8,42 (1H, s, NH); ^13^C NMR (75 Hz, DMSO-*d*_6_): *δ* 56.29, 112.31, 115.08, 117.71, 119.51, 120.81, 124.33, 129.82, 141.21, 149.81, 150.33, 162.02, 164.59;* m/z* = 331 (M^+^), 333 (M^+^+2). Cal/Ana: [C (57.98) 57.93 H (4.22) 4.25 N (12.56) 12.67].

#### 2.4.18. N-(4-Chlorophenyl)-5-(2-furyl)-1,3,4-oxadiazol-2-amine (**4r**)

Yield 78%, Mp. 108–112°C; IR: (KBr) cm^−1^: 3216 (NH), 1511 (C=N), 1119 (C–O–C), 694 (C–Cl). ^1^H NMR (300 MHz, DMSO-*d*_6_): *δ* 7.27–7.29 (2H, d,* J* = 6.0 Hz, ArH), 7.41–7.43 (2H, d,* J* = 6.0 Hz, ArH), 7.65–7.70 (3H, m, ArH), 8.47 (1H, s, NH);* m/z* = 261 (M^+^), 263 (M^+^+2). Cal/Ana: [C (55.03) 55.08 H (3.06) 3.08 N (16.07) 16.06].

#### 2.4.19. N-(2,4-Dimethylphenyl)-5-(4-methoxyphenyl)-1,3,4-oxadiazol-2-amine (**4s**)

Yield 72%, Mp. 180–182°C; IR: (KBr) cm^−1^: 3209 (NH), 1523 (C=N), 1171 (C–O–C). ^1^H NMR (300 MHz, DMSO-*d*_6_): *δ* 2.21 (6H, s, CH_3_), 3.78 (3H, s, OCH_3_), 6.95–6.97 (2H, t,* J* = 7.5 Hz, ArH), 7.25–7.27 (2H, d,* J* = 6.1 Hz, ArH), 7.12–7.14 (2H, d,* J* = 6.6 Hz, ArH), 7.62 (1H, s, ArH), 8.45 (1H, s, NH); ^13^C NMR (75 Hz, DMSO-*d*_6_): *δ* 15.88, 24.63, 56.23, 112.32, 115.81, 116.11, 118.53, 120.82, 126.93, 128.32, 128.92, 131.79, 139.11, 149.81, 150.32, 162.71, 164.55;* m/z* = 295 (M^+^). Cal/Ana: [C (69.12) 69.14 H (5.85) 5.80 N (14.19) 14.23].

#### 2.4.20. N-(2,4-Dimethylphenyl)-5-(4-fluorophenyl)-1,3,4-oxadiazol-2-amine (**4t**)

Yield 68%, Mp. 190–192°C; IR: (KBr) cm^−1^: 3191 (NH), 1521 (C=N), 1198 (C–O–C), 788 (C–F). ^1^H NMR (300 MHz, DMSO-*d*_6_): *δ* 2.23 (6H, s, CH_3_), 6.97-6.92 (2H, t,* J* = 7.5 Hz, ArH), 7.21–7.23 (2H, d,* J* = 6.3 Hz, ArH), 7.14–7.44 (2H, d,* J* = 6.6 Hz, ArH), 7.91 (1H, s, ArH), 8.45 (1H, s, NH);* m/z* = 283 (M^+^), 285 (M+2)^+^. Cal/Ana: [C (67.87) 67.83 H (4.91) 4.98 N (14.80) 14.83].

#### 2.4.21. N-(2,4-Dimethylphenyl)-5-(4-chlorophenyl)-1,3,4-oxadiazol-2-amine (**4u**)

Yield 68%, Mp. 204–206°C; IR: (KBr) cm^−1^: 3192 (NH), 1539 (C=N), 1173 (C–O–C), 697 (C–Cl). ^1^H NMR (300 MHz, DMSO-*d*_6_): *δ* 2.49 (6H, s, CH_3_), 6.39–7.41 (7H, t,* J* = 8.7 Hz, ArH), 7.31–7.34 (2H, d,* J* = 8.7 Hz, ArH), 7.67–7.70 (2H, d,* J* = 8.7 Hz, ArH), 7.95 (1H, s, ArH), 8.98 (1H, s, NH);* m/z* = 299 (M^+^), 301 (M+2)^+^. Cal/Ana: [C (64.05) 64.11 H (4.67) 4.71 N (14.01) 14.02].

#### 2.4.22. N-(2,4-Dimethylphenyl)-5-(4-hydroxyphenyl)-1,3,4-oxadiazol-2-amine (**4v**)

Yield 74%, Mp. 200–202°C; IR: (KBr) cm^−1^: 3412 (OH), 3197 (NH), 1511 (C=N), 1179 (C–O–C). ^1^H NMR (300 MHz, DMSO-*d*_6_): *δ* 2.22 (6H, s, CH_3_), 6.99–7.01 (2H, t,* J* = 7.5 Hz, ArH), 7.20–7.22 (2H, d,* J* = 6.3 Hz, ArH), 7.41–7.43 (2H, d,* J* = 6.6 Hz, ArH), 7.66 (1H, s, ArH), 8.45 (1H, s, NH); 10.62 (1H, s, OH);* m/z* = 281 (M^+^). Cal/Ana: [C (68.25) 68.31 H (5.29) 5.37 N (14.99) 14.94].

#### 2.4.23. N-(2,4-Dimethylphenyl)-5-(3,4-dimethoxyphenyl)-1,3,4-oxadiazol-2-amine (**4w**)

Yield 70%, Mp. 178–180°C; IR: (KBr) cm^−1^: 3202 (NH), 1521 (C=N), 1139 (C–O–C). ^1^H NMR (300 MHz, DMSO-*d*_6_): *δ* 2.23 (6H, s, CH_3_), 3.79 (6H, s, OCH_3_), 6.94–6.97 (2H, d,* J* = 8.4 Hz, ArH), 7.12–7.14 (1H, d,* J* = 7.1 Hz, ArH), 7.47 (1H, s, ArH), 7.54–7.56 (1H, d,* J* = 6 Hz, ArH), 7.85 (1H, m, ArH), 8.40 (1H, s, NH); ^13^C NMR (75 Hz, DMSO-*d*_6_): *δ* 15.83, 24.67, 56.23, 112.31, 115.89, 116.16, 118.59, 120.81, 126.99, 128.30, 128.92, 131.70, 139.19, 149.83, 150.32, 162.71, 164.55;* m/z* = 325 (M^+^). Cal/Ana: [C (66.41) 66.45 H (5.86) 5.89 N (12.86) 12.91].

#### 2.4.24. N-(2,4-Dimethylphenyl)-5-(2-furyl)-1,3,4-oxadiazol-2-amine (**4x**)

Yield 66%, Mp. 190–192°C; IR: (KBr) cm^−1^: 3219 (NH), 1523 (C=N), 1109 (C–O–C). ^1^H NMR (300 MHz, DMSO-*d*_6_): *δ* 2.23 (6H, s, CH_3_), 6.91–6.93 (2H, t,* J* = 7.5 Hz, ArH), 7.25–7.27 (2H, d,* J* = 6.1 Hz, ArH), 7.34–7.36 (2H, d,* J* = 6.6 Hz, ArH), 7.79 (1H, s, ArH), 8.41 (1H, s, NH);* m/z* = 255 (M^+^). Cal/Ana: [C (65.76) 65.87 H (5.09) 5.13 N (16.52) 16.46].

### 2.5. Anticancer Activity

The compounds were submitted to the National Cancer Institute (NCI US) and were screened on NCI 60 cell lines initially at a single high dose (10^−5^ M) on leukemia, melanoma, lung, colon, CNS, ovarian, renal, prostate, and breast cancers cell lines. The one-dose data were reported as a mean graph of the percent growth (GP) of treated cells. The number reported for the one-dose assay is growth relative to the no-drug control and relative to the time zero number of cells. The anticancer screening was carried out as per the NCI US protocol reported elsewhere [24–27]. We have discussed the anticancer screening method in our previous work [6, 28].

## 3. Results and Discussions

### 3.1. Chemistry

**Scheme 1:**
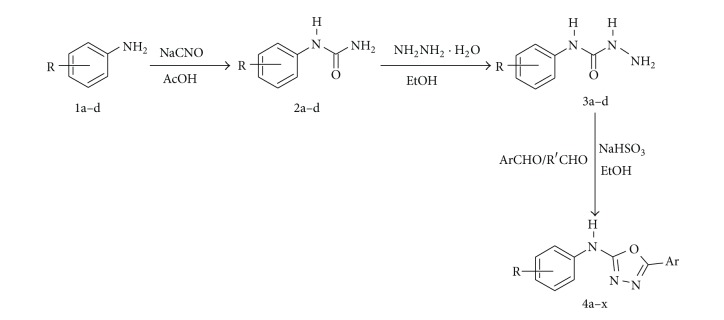
Protocol for the synthesis of 5-substituted-*N*-aryl-1,3,4-oxadiazol-2-amine analogues (**4a–x**).

In the first step, aromatic anilines (**1a–d**) were treated with sodium cyanate in glacial acetic acid to obtain substituted phenyl ureas (**2a–d**) which was then treated with hydrazine hydrate to obtain substituted phenyl semicarbazides (**3a–d**). In the final step, substituted phenyl semicarbazides (**3a–d**) and aromatic aldehydes were refluxed for 12–14 h using 20 mol% NaHSO_3_ and ethanol-water system (1 : 2, v/v) solvent to obtain oxadiazole analogues (**4a–x**). The reaction was monitored throughout by thin layer chromatography (TLC) using chloroform: methanol (9 : 1) and acetone : n-hexane (8 : 2) as mobile phase, and the purity of the compounds was checked by elemental analysis. The reaction sequence is shown in Scheme 1. The synthesized compounds were characterized by spectral analysis, and all the compounds were in full harmony with the proposed structures. In general, the IR spectra afforded absorption 3191–3222 cm^−1^ band due to NH band, 1511–1531 cm^−1^ band due to C=N, and 1109–1203 cm^−1^ band due to oxadiazole stretching. In ^1^H NMR the signals of the respective protons of the synthesized title compounds were verified on the basis of their chemical shifts and multiplicities in DMSO* d*_*6*_. The spectra showed a triplet at *δ* 1.32–1.34 ppm corresponding to CH_3_; a singlet at *δ* 2.22–2.37 ppm corresponding to aromatic CH_3_; a singlet at 3.73–3.79 ppm corresponding to OCH_3_; a singlet at *δ* 8.05–8.95 ppm corresponding to NH; singlet, doublets, triplet, and multiplet at *δ* 6.71–7.95 ppm corresponding to aromatic protons; and a singlet at *δ* 10.36–10.62 ppm corresponding to OH.

### 3.2. Anticancer Activity

All compounds submitted to the NCI 60 cell screen were tested initially at a single high dose (10^−5^ M) on leukemia, melanoma, lung, colon, CNS, ovarian, renal, prostate, and breast cancers cell lines, nearly 60 in number. Compound* N*-(2,4-dimethylphenyl)-5-(4-methoxyphenyl)-1,3,4-oxadiazol-2-amine (**4s**) showed maximum activity with mean growth percent (GP) of 62.62 followed by* N*-(2,4-dimethylphenyl)-5-(4-chlorophenyl)-1,3,4-oxadiazol-2-amine (**4u**) with mean GP of 78.46 while rest of the compounds showed less mean GP of more than 97.03. The compound** 4s** was highly active on MDA-MB-435 (melanoma) [GP = 15.43], K-562 (leukemia) [GP = 18.22], T-47D (breast cancer) [GP = 34.27], and HCT-15 (colon cancer) [GP = 39.77]. The compound** 4u** showed maximum activity on MDA-MB-435 (melanoma) [GP = 6.82], K-562 (leukemia) [GP = 24.80], NCI-H522 (non-small-cell lung cancer) [GP = 41.03], and HCT (colon cancer) [GP = 44.74].* N*-(4-Bromophenyl)-5-(3,4-dimethoxyphenyl)-1,3,4-oxadiazol-2-amine (**4j**) showed anticancer activity with GP of 75.06 (HOP-92; non-small-cell lung cancer), 76.31 (MOLT-4; leukemia), 79.42 (NCI-H522; non-small-cell lung cancer), and 81,73 (SNB-75; CNS cancer).* N*-(4-Bromophenyl)-5-ethyl-1,3,4-oxadiazol-2-amine (**4l**) showed GP of 76.62 (A498; renal cancer), 77.96 (MALME-3M; melanoma), and 79.51 (MOLT-4; leukemia).* N*-(4-Bromophenyl)-5-(4-chlorophenyl)-1,3,4-oxadiazol-2-amine (**4h**) showed GP of 82.94 (SR; leukemia), 60.45 (SK-MEL-2; melanoma), 67.42 (MDA-MB-231/ATCC; breast cancer), 80.02 (UO-31; renal cancer), and 82.97 (MCF7; breast cancer). Rest of the compounds had less average GP albeit showing good activity against some cell lines, namely, compound** 4f** [GP = 65.75; SR (leukemia)], compound** 4a** [GP = 72.88; NCI-H522 (non-small-cell lung cancer)], and compound** 4c** [GP = 76.67; SR (leukemia)]. The maximum activity was observed on MDA-MB-435 (melanoma) with GP of 6.32 while rest of the compounds showed GP of >59.21. The anticancer activity of the compounds is given in Table 3. The structure activity relationship obtained from the screening results showed that* N*-aryl with 2,4-dimethyl substitution was more promising than methyl substitution and 4-dimethoxyphenyl, 3,4-dimethoxyphenyl, and ethyl substitution at position 5 of oxadiazole showed more activity.

## 4. Conclusion

A series of newer oxadiazole analogues were subjected to molecular properties prediction by Molinspiration and toxicity risk prediction by Osiris software and synthesized in satisfactory yields. All the compounds followed the Lipinski rule of five which makes them potentially active agents and were also found to be less toxic than the standard anticancer drug methotrexate and fluorouracil (as per Osiris prediction). 16 compounds were evaluated for their anticancer activity in one-dose assay and showed moderate activity on various cell lines.* N*-(2,4-Dimethylphenyl)-5-(4-methoxyphenyl)-1,3,4-oxadiazol-2-amine could be considered as lead for further discovery and could be modified to potentiate the anticancer activity. Further studies to acquire more information about quantitative structure activity relationships (QSAR) and molecular docking studies are currently in progress in our laboratory.

## Figures and Tables

**Table 1:** Pharmacokinetic parameters important for good oral bioavailability of *N*-aryl-5-substituted-1,3,4-oxadiazol-2-amine analogues (**4a–x**).

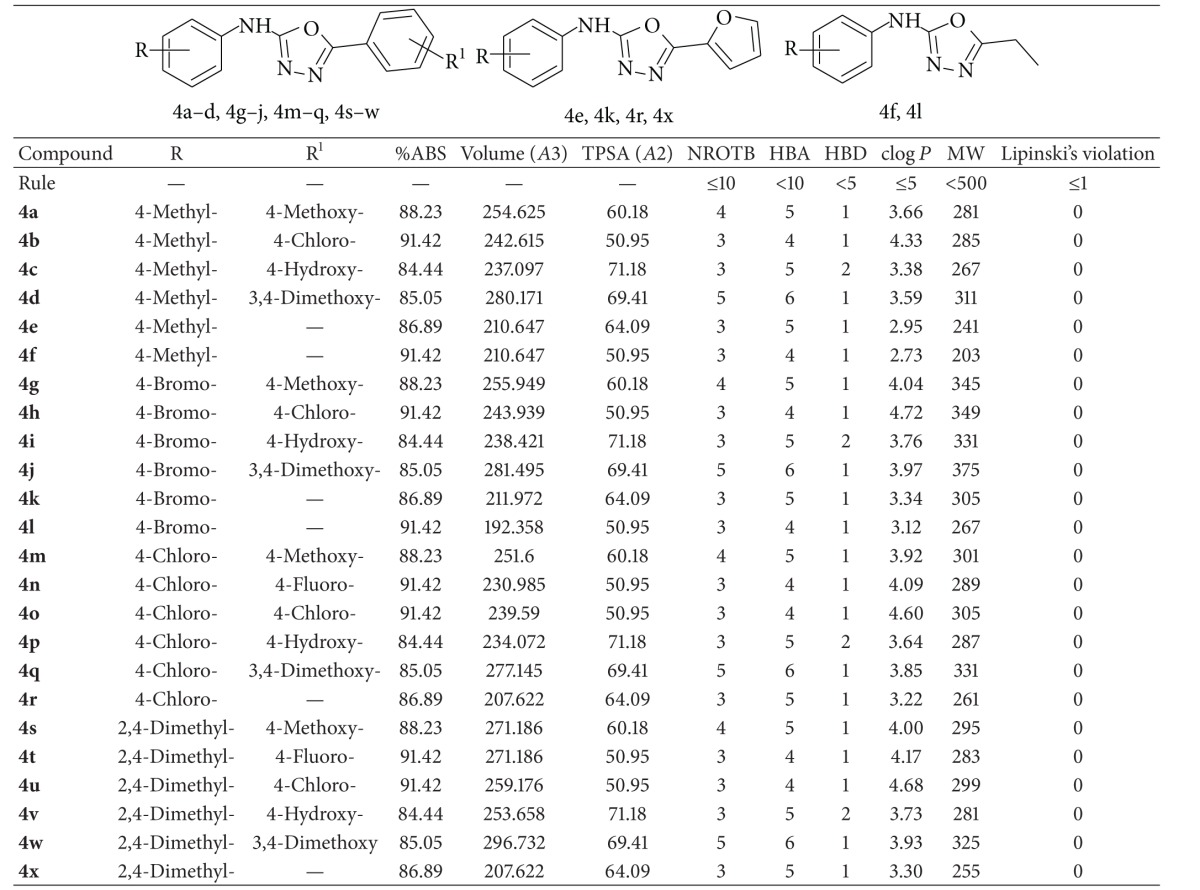

%ABS: percentage of absorption; TPSA: topological polar surface area; NROTB: number of rotatable bonds; MW: molecular weight; Log *P*: logarithm of compound partition coefficient between n-octanol and water; HBD: number of hydrogen bond donors; HBA: number of hydrogen bond acceptors.

**Table 2:** Prediction of toxicity risk of the *N*-aryl-5-substituted-1,3,4-oxadiazol-2-amine analogues (**4a–x**).

Compound	Prediction of toxicity risk by Osiris		
	MUT	IRRI	REP
**4a**	—	—	—
**4b**	—	—	—
**4c**	—	—	—
**4d**	—	—	—
**4e**	+	—	—
**4f**	—	—	+
**4g**	—	—	—
**4h**	—	—	—
**4i**	—	—	—
**4j**	—	—	—
**4k**	+	—	—
**4l**	—	—	+
**4m**	—	—	—
**4n**	—	—	—
**4o**	—	—	—
**4p**	—	—	—
**4q**	+	—	—
**4r**	—	—	—
**4s**	—	+^a^	—
**4t**	—	+^a^	—
**4u**	—	+^a^	—
**4v**	—	+^a^	—
**4w**	—	+^a^	—
**4x**	—	+^a^	—
Methotrexate	+^a^	—	+^a^
Fluorouracil	+	+	+

MUT: mutagenic, IRRI: irritant, REP: reproductive effect. A dash (—) indicates no effect, a plus (+) indicates the effect, and (+^a^) indicates slight effect.

**Table 3:** *In vitro* anticancer activity of *N*-aryl-5-substituted-1,3,4-oxadiazol-2-amine analogues (**4a–x**).

Compound	60 cell lines assay in one dose 10^−5^ M conc.				
	NSC Code	Mean GP	Range of GP	The most sensitive cell lines	GP of the most sensitive cell lines
**4a**	776721	98.88	72.88 to 114.64	NCI-H522 (non-small-cell lung cancer) SNB-75 (CNS cancer) MCF7 (breast cancer) A498 (renal cancer)	72.88 80.44 83.98 85.88

**4b**	776720	102.09	84.59 to 123.42	A498 (renal cancer) T-47D (breast cancer) UO-31 (renal cancer) MCF7 (breast cancer)	84.59 86.46 92.10 92.37

**4c**	777952	98.55	76.67 to 117.71	SR (leukemia) K-562 (leukemia) HL-60 (TB) (leukemia) SNB-75 (CNS cancer)	76.67 81.05 82.02 83.85

**4d**	776719	100.71	83.46 to 127.18	UO-31 (renal cancer) MDA-MB-231/ATCC (breast cancer) SK-OV-3 (ovarian cancer) MCF7 (breast cancer)	83.46 86.63 89.42 89.92

**4e**	776722	100.59	80.87 to 117.08	HOP-92 (non-small-cell lung cancer) UO-31 (renal cancer) HL-60 (TB) (leukemia) NCI-H522 (non-small-cell lung cancer)	80.87 83.10 87.92 88.13

**4f**	777951	98.50	65.75 to 110.26	SR (leukemia) MOLT-4 (leukemia) UO-31 (renal cancer) HCT-116 (colon cancer)	65.75 82.58 87.20 88.20

**4h**	776724	97.30	60.45 to 111.98	SK-MEL-2 (melanoma) MDA-MB-231/ATCC (breast cancer) UO-31 (renal cancer) MCF7 (breast cancer)	60.45 67.42 80.02 82.97

**4i**	777954	97.93	75.33 to 118.40	HL-60 (TB) (leukemia) K-562 (leukemia) SR (leukemia) NCI-H522 (non-small-cell lung cancer)	75.33 81.88 85.63 88.68

**4j**	776723	97.03	75.06 to 120.27	HOP-92 (non-small-cell lung cancer) MOLT-4 (leukemia) NCI-H522 (non-small-cell lung cancer) SNB-75 (CNS cancer)	75.06 76.31 79.42 81.73

**4k**	776725	97.80	73.29 to 116.30	PC-3 (prostate cancer) UO-31 (renal cancer) MOLT-4 (leukemia) HOP-92 (non-small-cell lung cancer)	73.29 82.21 83.75 84.14

**4l**	777953	97.10	76.62 to 112.24	A498 (renal cancer) MALME-3M (melanoma) MOLT-4 (leukemia) SR (leukemia)	76.62 77.96 79.51 82.94

**4m**	776715	101.09	79.80 to 128.96	A498 (renal cancer) SK-MEL-2 (melanoma) HL-60 (TB) (leukemia) MCF7 (breast cancer)	79.80 80.78 80.81 81.12

**4n**	776716	100.42	59.21 to 116.24	SK-MEL-2 (melanoma) UO-31 (renal cancer) MOLT-4 (leukemia) BT-549 (breast cancer)	59.21 82.84 84.21 86.27

**4s**	777948	**62.61**	**15.43 to 88.49**	MDA-MB-435 (melanoma) K-562 (leukemia) T-47D (breast cancer) HCT-15 (colon cancer)	**15.43** **18.22** **34.27** **39.77**

**4u**	777949	**78.46**	**6.82 to 106.57**	MDA-MB-435 (melanoma) K-562 (leukemia) NCI-H522 (non-small-cell lung cancer) HCT-15 (colon cancer)	**6.82** **24.80** **41.03** **44.74**

**4w**	777950	101.29	80.97 to 115.23	T-47D (breast cancer) A498 (renal cancer) HCT-116 (colon cancer) UO-31 (renal cancer)	80.97 87.44 89.01 90.55
